# Identification of changes in bile composition in pancreaticobiliary reflux based on liquid chromatography/mass spectrometry metabolomics

**DOI:** 10.1186/s12876-023-03097-4

**Published:** 2024-01-02

**Authors:** Xuanbo Da, Yukai Xiang, Hai Hu, Xiangyu Kong, Chen Qiu, Zhaoyan Jiang, Gang Zhao, Jingli Cai, Anhua Huang, Cheng Zhang, Chuanqi He, Beining Lv, Honglei Zhang, Yulong Yang

**Affiliations:** Center of Gallbladder Disease, Shanghai East Hospital, Institute of Gallstone Disease, School of Medicine, Tongji University, Shanghai, 200092 China

**Keywords:** Pancreaticobiliary reflux, Bile, Gallstones, Liquid chromatography–mass spectrometry metabolomics

## Abstract

**Introduction:**

Pancreaticobiliary reflux (PBR) can induce gallstone formation; however, its pathogenic mechanism remains unclear. In this study, we explored the mechanism of PBR by the non-targeted metabolomic analysis of bile in patients with PBR.

**Objective:**

The aim of this study was to investigate the pathogenic mechanism in PBR by the non-targeted metabolomic analysis of bile collected during surgery.

**Methods:**

Sixty patients who underwent gallstone surgery at our center from December 2020 to May 2021 were enrolled in the study. According to the level of bile amylase, 30 patients with increased bile amylase ( > 110 U/L) were classified into the PBR group, and the remaining 30 patients were classified into the control group (≤ 110 U/L). The metabolomic analysis of bile was performed.

**Results:**

The orthogonal projections to latent structure-discriminant analysis of liquid chromatography mass spectrometry showed significant differences in bile components between the PBR and control groups, and 40 metabolites were screened by variable importance for the projection value (VIP > 1). The levels of phosphatidylcholine (PC) and PC (20:3(8Z,11Z,14Z)/14:0) decreased significantly, whereas the levels of lysoPC (16:1(9z)/0:0), lysoPC (15:0), lysoPC (16:0), palmitic acid, arachidonic acid, leucine, methionine, L-tyrosine, and phenylalanine increased.

**Conclusions:**

Significant differences in bile metabolites were observed between the PBR and control groups. Changes in amino acids and lipid metabolites may be related to stone formation and mucosal inflammation.

**Supplementary Information:**

The online version contains supplementary material available at 10.1186/s12876-023-03097-4.

## Introduction

Most of the knowledge about epithelial injury, hyperplasia, metaplasia, and gallbladder and bile duct cancer related to pancreaticobiliary reflux (PBR) is based on the study of pancreaticobiliary maljunction (PBM). Recently, some scholars have discussed a phenomenon of PBR, occult PBR, which occurs in a normal pancreaticobiliary junction, and it may play a role in gallstone formation by damaging the gallbladder mucosa [1–3].

Many clinical studies have focused on PBR pathogenesis. Current research shows that continuous PBR causes chronic inflammation and injury to the biliary tract mucosa and the gallbladder mucosa. When bile and pancreatic enzyme accumulation reaches a high level, acute and chronic cholecystitis, common bile duct stones, and gallbladder polyps can develop [2]. The reflux of pancreatic juice damages the contraction function of the gallbladder, which leads to changes in bile components, promotes the secretion of mucus proteins, and finally forms bile mud and gallstones [4–6]. The currently recognized carcinogenic mechanism of PBR-related biliary tumors is via phospholipase A2 (PLA2), which hydrolyzes lecithin into lysophosphatidylcholine (lysoPC), damaging the biliary epithelium [7], thereby leading to chronic gallbladder inflammation and stone formation. The combined effect of this inflammation and stone formation promotes the occurrence of gallbladder cancer [8, 9]. The microsatellite instability and mRNA index of tumor suppressor gene mutations increased significantly in the biliary epithelium of patients with PBR, indicating that pancreatic juice reflux induced the production of mutagenic metabolites and promoted epithelial cell carcinogenesis [5, 7]. However, the mechanism by which PBR causes gallstone formation remains unclear.

Metabolomics is a newly developed discipline after genomics and proteomics, which simultaneously performs the qualitative and quantitative analysis of all low-molecular-weight metabolites in an organism during a specific physiological period [8]. This new technology is one of the effective means to understand the pathophysiology of various diseases, disease diagnosis, and biomarkers, which can help find new biomarkers, discover new metabolic pathways or better understand currently known metabolic pathways [8, 10, 11]. Bile metabolism plays a key role in gallstone disease pathogenesis. However, no study on bile metabolomics in patients with PBR is available.

Here, liquid chromatography–mass spectrometry (LC–MS) metabolomics was used for the first time to extensively analyze changes in bile sample components in patients with and without PBR. We identified potential differential bile metabolites, which might provide a theoretical basis for elucidating the lithogenic mechanism of PBR.

## Patients and methods

### Patients

This prospective study continuously included patients who underwent surgery for gallstones due to biliary colic between December 2020 and May 2021 at our hospital.

Here are the exclusion criteria based on preoperative assessment: (1) acute cholecystitis, (2) cholangitis, (3) acute or chronic pancreatic disease, (4) abnormal serum amylase and lipase values, (5) common bile duct stones, (6) had undergone preoperative endoscopic cholangiography or sphincterotomy, and (7) inability to obtain written informed consent. Here are the exclusion criteria based on intraoperative assessment: (1) the gallbladder filled with thick sticky bile, (2) the small shrunken and atrophic gallbladder, (3) the gallbladder with no bile content at all, and (4) the cystic duct was occluded, and the gallbladder was hydropic.

### Processing and analysis of bile samples

During the operation, bile (5 mL) was obtained from the gallbladder using a syringe, 1 mL was stored in a sterile tube at 4 °C, whereas the rest of it was immediately preserved at − 80 °C for sample preparation and analysis and was sent to the laboratory on the next day. All samples were processed and measured by laboratory technicians using Roche Cobas c702 (Roche Diagnostics, Basel, Switzerland), and the technicians were not informed of the study and source of the samples. The normal value of serum amylase is 30–110 U/L.

The origin of amylase in bile is generally attributed to serum amylase, which passes through the liver, as well as reflux from the pancreatic duct [12]. However, there is currently no universally accepted standard for what constitutes a ‘normal’ level of amylase in bile. In their seminal work, Donaldson et al. [12] carried out intraoperative sampling of bile in patients without liver disease whose serum hepatobiliary enzyme levels were normal, and analysis of the bile revealed no difference between the amylase level in bile and the serum amylase level. Consistent with the methodologies used in other relevant studies [2, 13–15], this study classifies bile amylase levels that surpass the established normal plasma thresholds as indicative of PBR [2, 14, 16, 17]. In this study, 30 patients with gallstones combined with PBR and 30 patients without PBR were included. The control group consisted of patients with gallstones combined without PBR.

### Metabolite extraction

A total of 100 µL of each sample was transferred to an Eppendorf tube. After adding the 400 µL of an extract solution (acetonitrile:methanol = 1:1, containing isotopically-labeled internal standard mixture), the samples were vortexed for 30 s, sonicated for 10 min in an ice-water bath, and incubated for 1 h at − 40 °C to precipitate proteins. Then the samples were centrifuged at 12,000 rpm (RCF = 13,800 × *g*, R = 8.6 cm) for 15 min at 4 °C. The resulting supernatants were transferred to a fresh glass vial for further analysis. A quality control sample was prepared by mixing an equal aliquot of the supernatants of all samples.

### Metabolic profiling of bile

LC–MS/MS analyses were performed using a UHPLC system (Vanquish, Thermo Fisher Scientific) with the UPLC BEH Amide column (2.1 mm × 100 mm, 1.7 μm) coupled with the Q Exactive HFX mass spectrometer (Orbitrap MS, Thermo). The mobile phase consisted of 25 mmol/L ammonium acetate and 25 ammonia hydroxide in water (pH = 9.75) (A) and acetonitrile (B). The auto-sampler temperature was 4 °C, and the injection volume was 2 µL. The QE HFX mass spectrometer was used for its ability to acquire MS/MS spectra on information-dependent acquisition mode in the control of an acquisition software (Xcalibur, Thermo). In this mode, the acquisition software continuously evaluates the full scan MS spectrum. The ESI source conditions were set as follows: sheath gas flow rate, 30 Arb; Aux gas flow rate, 25 Arb; capillary temperature, 350 °C; full MS resolution, 60,000; MS/MS resolution, 7500; collision energy, 10/30/60 in NCE mode; and spray voltage, 3.6 kV (positive) or − 3.2 kV (negative).

### Bioinformatics and statistical analysis

Simca-p 14.1 (Umetrics, Umea, Sweden) was used for pattern recognition. After the data were preprocessed by Pareto scaling, multivariate statistical analysis was performed, including unsupervised principal component analysis (PCA) and orthogonal projections to latent structure-discriminant analysis (OPLS-DA). To perform this analysis, we calculated the variable importance for the projection (VIP) value and used VIP > 1.0 as a screening criterion for differential metabolites. Moreover, commercial databases, including the Kyoto Encyclopedia of Genes and Genomes (http://www.genome.jp/kegg/), were used to search for pathways of bile metabolites. For all analyses, *P* < 0.05 was considered statistically significant.

## Results

### Characteristics of the study population

No signifcant diferences were found between the groups with regard to age, gender, BMI, characteristics of gallbladder stones: cholesterol / mixed / pigmented, comorbidity: diabetes, hypertension, hypercholesterolemia, hypertriglyceridemia(Table 1). The bile amylase level in the PBR group was significantly higher than that in the Control group (Supplementary Fig. 1).

**Table 1:** Clinical characteristics of gallstone patients with or without PBR

Variables	C group (n = 30)	PBR group (n = 30)	*P*-value
Gender (M/F)	18/30	19/30	NS
Age (years), median (Q1 - Q3)	54.5 (41.75-63)	57.5 (50.75-65)	NS
BMI (kg*m − 2), median (Q1 - Q3)	24 (21–26)	23 (20.75-25)	NS
Hypertension, n (%)	30%	23.33%	NS
hypercholesterolemia, n (%)	0	3.33%	NS
hypertriglyceridemia, n (%)	10.00%	6.67%	NS
Diabetes history, n (%)	6.67%	3.33%	NS
Amylase levels (U/L), median (Q1 - Q3)	30 (10-52.5)	2520 (975-17500)	< 0.001
characteristics of gallbladder stones,n (%)			
cholesterol	86.67%	83.33%	NS
mixed	6.67%	10.00%	NS
pigmented	6.67%	6.67%	NS

Data are represented as the median (25th − 75th percentiles) or percentage

C: control, PBR: pancreaticobiliary reflux, M: male, F: female, BMI: body mass index, NS: no significance

### Multivariate analysis of bile metabolites

**Fig. 1:**
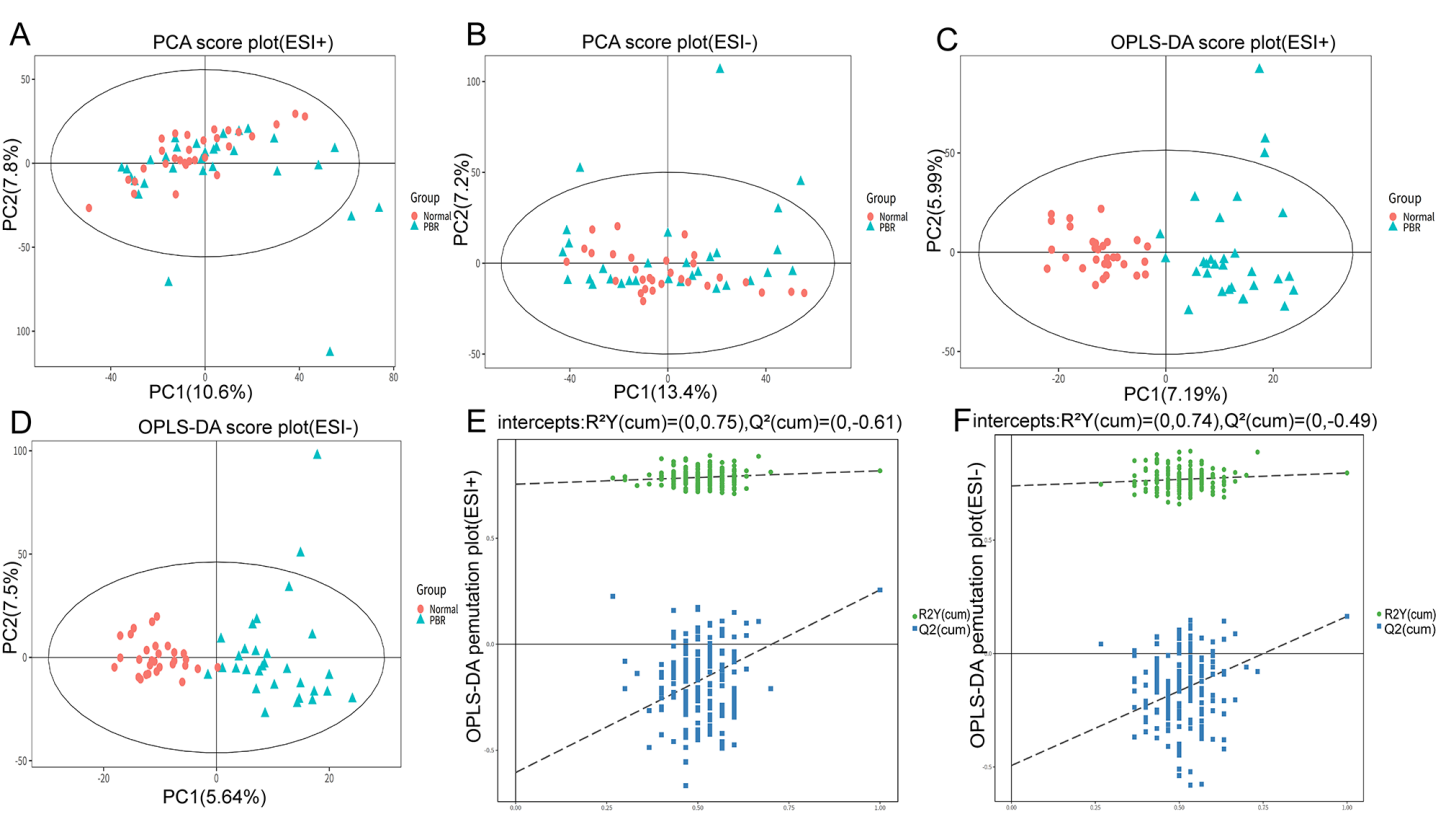
Principal component analysis score plots (**A** and **B**), Orthogonal projections to latent structure-discriminate analysis (OPLS-DA) score plots (**C** and **D**), and permutation tests of the OPLS-DA mode (**E** and **F**) of bile metabolomic analysis

The bile samples were characterized by LC–MS in the positive and negative ion modes to obtain the mass spectra of the two groups of the bile samples. The mass spectral data were then processed by multivariate analysis, which mainly included PCA and OPLS-DA, to observe the stability of the whole analysis and distribution between the samples. PCA was initially conducted to generate an outline of the bile metabolites variabilities between patients with PBR, and controls. The PCA score chart showed that the samples were within Hotelling’s t-squared ellipse, and a certain separation trend was observed between the sample data obtained under the positive and negative ion modes, indicating that changes in some bile metabolites were related to disease processes (Fig. 1A, B).

Moreover, we established an OPLS-DA model based on positive and negative ion mode data to further verify the separation trend of the metabolic spectrum between the two groups. The results revealed a separation trend between the PBR and benign biliary tract disease groups (Fig. 1C, D). As a key model for differential metabolite screening, the robustness of OPLS-DA model will significantly affect the key conclusions of the entire data analysis. Therefore, we conducted an additional evaluation of the robustness of OPLS-DA model. The permutation test of the OPLS-DA model showed the original model R^2^Y is close to 1, indicating that the model is more in line with the real situation of sample data. Also the permutation test demonstrating that the model was well‑fit and the modes had good explanatory and predictive capabilities (Fig. 1E, F).

### Screening and identification of differential metabolites

For analyzing differential metabolites classified in the two groups of the samples, we considered VIP values greater than 1 as a screening criterion for these differential metabolites. After material identification, 40 metabolites showed reliable results (Supplementary Table 1). Among these differential metabolites, the levels of four metabolites decreased, whereas the levels of the remaining 36 metabolites increased significantly compared with those in the control group. These metabolites mainly included amino acids and lipid compounds. In the PBR group, the levels of PC and PC (20:3 (8Z, 11z, 14z)/14:0) decreased significantly, whereas the levels of lysoPC, palmitic acid, and arachidonic acid increased significantly. Moreover, an increase in the levels of palmitoleic acid and arachidonic acid was observed. The levels of various amino acids, including leucine, methionine, and phenylalanine, increased significantly in the PBR group.

### Pathway enrichment and metabolic pathway analysis of the potential metabolic mechanism

**Fig. 2:**
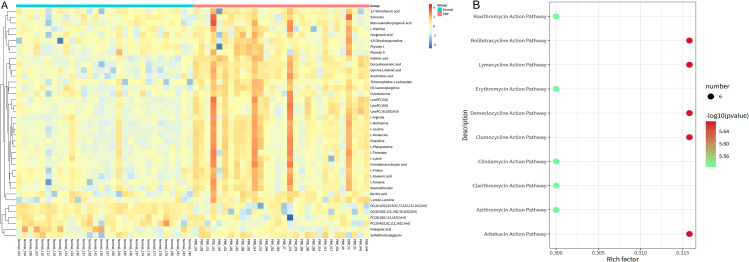
Potential metabolic pathways in patients with pancreaticobiliary reflux (PBR). **(A)** Hierarchical cluster analysis heatmap of the bile metabolic profiles of the PBR and control groups. Red represents upregulation, whereas blue represents downregulation. Each column represents an individual sample, and each row represents a compound. **(B)** Bubble plot of the potential metabolic pathways in the PBR group

We used the levels of qualitatively significant differential metabolites to perform the hierarchical clustering of the samples in the two groups. The heatmap showed differences between the metabolic profiles of the samples in the two groups (Fig. 2A). Pathway enrichment analysis was performed to identify affected metabolic and signal transduction pathways after PBR. The pathway with an impact-value threshold above 0.10 was considered the potential target pathway [18]. As shown in Fig. 2B, protein digestion and absorption, mineral absorption, lysine degradation, linoleic acid metadata, D-amino acid metadata, central carbon metadata in cancer, arginine and proline metadata, aminoacyl-tRNA biosynthesis, alanine, aspartate, and glutamate metadata, and ABC transporters were significantly different between the two groups. The matching status, *P*-value, −log10 (*P*-value), and rich factor of each pathway are presented in Table 2, which indicates that the metabolic pathways of lipids and some amino acids changed significantly after PBR.

**Table 2:** Results of pathway enrichment analysis of signifcant metabolites

No.	Pathway	Total	Hits number	Raw p	-log10(p)	Rich factor
1	D-Amino acid metabolism	40	15	9.88538E-17	16.0050	0.224
2	Protein digestion and absorption	40	13	7.66515E-16	15.1154	0.277
3	Central carbon metabolism in cancer	40	11	7.23559E-14	13.1405	0.297
4	Aminoacyl-tRNA biosynthesis	40	12	1.32113E-13	12.8790	0.231
5	Mineral absorption	40	7	1.98608E-08	7.70200	0.241
6	ABC transporters	40	11	2.38156E-07	6.62314	0.079
7	Arginine and proline metabolism	40	6	0.0001193	3.92311	0.086
8	Lysine degradation	40	5	0.0002249	3.64786	0.1
9	Alanine, aspartate and glutamate metabolism	40	4	0.0002512	3.59984	0.143
10	Linoleic acid metabolism	40	4	0.0002512	3.59984	0.143

## Discussion

PBR is closely related to the occurrence and development of gallstones [19, 20]. Long-term PBR can cause chronic inflammation of the gallbladder mucosa and changes in bile composition and ultimately induce the formation of gallstones [3, 17]. Chronic inflammatory changes in the gallbladder mucosa are often accompanied by decreased gallbladder motility and changes in bile transport, absorption, and secretion, which can induce gallstone formation [21, 22]. Moreover, increased PLA2 levels affect the formation of bile salt-lecithin microparticles, affecting the dissolution of cholesterol, thereby leading to gallstone formation [21, 23]. However, the mechanism of PBR that leads to gallstone formation is still unclear, and new methods are needed to diagnose PBR. Here, we used the LC–MS method to perform the metabolomic analysis of the bile samples of 60 patients.

Some scholars have reported that metabolomic analysis can be a new method for diagnosing breast cancer, biliary tract cancer, and PBM [24, 25]. Previous results showed that the bile metabolites of patients with PBM and extrahepatic cholangiocarcinoma were similar, and significant differences were observed between the control and PBM or extrahepatic cholangiocarcinoma groups. Previously, bile metabolomic methods were only used for diagnosing bile duct cancer and PBM, and few studies are available on their application [25, 26]. Here, we analyzed many samples. The metabolomic analysis results showed that the OPLS-DA model established based on the positive and negative ion mode data obtained in this study was a good fit and statistically valid [25, 26]. The PCA and OPLS-DA models showed sufficient sensitivity and specificity to distinguish the PBR group from the control group. Compared with the control group, the levels of 106 bile metabolites related to energy homeostasis, amino acid metabolism, bile acid metabolism, and lipid synthesis changed significantly in the PBR group. Additionally, potential biomarkers that greatly contributed to this differentiation were identified and selected based on their VIP values for further study. The results showed that the levels of PC and PC (20:3 (8Z, 11z, 14z)/14:0) were significantly lower in the PBR group than in the control group, whereas the levels of lysoPC (16:1 (9z)/0:0), lysoPC (15:0), lysoPC (16:0), palmitic acid, leucine, methionine, L-tyrosine, and phenylalanine were significantly higher in the PBR group than in the control group. A strong correlation between the formation of gallstones and the high level of amylase in the gallbladder bile of patients with PBR has been observed [2, 15, 27], and PBR is usually caused by the reflux of pancreatic juice to the biliary tract, resulting in metabolic disorders of bile compounds, especially amino acid and lipid metabolism disorders, which lead to chronic inflammation and biliary mucosal damage [19, 25].

Many studies have shown that disorders of lipid profiles play a pivotal pathogenetic role in the initiation and progression of gallstones and gallbladder carcinoma (GBC) [25, 28]. LysoPC is one of the major lysophospholipids and is mainly generated by PC hydrolysis. PC is synthesized in liver cells, and its main function in bile is to form mixed micelles with bile acids and cholesterol to improve cholesterol solubility, which is important to ensure the stability of mixed micelles. PC also exerts cytoprotective effects and reduces the damage of the biliary epithelium caused by bile acids [29, 30]. After pancreatic juice reflux in the biliary tract, activated PLA2 hydrolyzes PC, resulting in decreased PC content in bile [17], which contributes to gallstone formation [31, 32]. LysoPC is mainly produced by the hydrolysis of PC by PLA2 [23]. LysoPC exhibits cytotoxicity by inducing biliary epithelial cell injury, and ultimately causing gallstone formation and biliary cancer [33]. Lyso-PC can increase the secretion of gallbladder mucin in cats and other animals and promote the formation of stones [34, 35]. Here, compared with the control group, PC in the bile of the PBR group decreased, and lysoPC increased significantly. Therefore, we speculate that Lyso-PC plays a role in promoting the formation of gallstones and the occurrence of biliary tumors in patients with PBR. Patients with biliary tract cancer were not included in this study. Therefore, a subsequent comparison of bile samples from patients with biliary tract cancer and those with simple gallstones are needed to verify our hypothesis.

It is believed that changes in bile composition caused by various reasons, including changes in cholesterol, phospholipids, and free fatty acids (FFA), are one of the main reasons for gallstone formation [36, 37]. Bile FFAs play an important role in maintaining the stable dissolution of cholesterol, and palmitic acid is the main component in this process [38]. Some studies have revealed that the total FFA content in the bile of patients with gallstones is significantly higher than that in normal individuals. Moreover, many unsaturated FFAs can damage the gallbladder mucosa and gallbladder contraction function and promote the high secretion of gallbladder mucin. All these factors play a positive role in gallstone formation [38]. Previous study showed that the total FFA content in the bile of patients with PBR was significantly higher than that in the control group [39]. Interestingly, here we found that palmitic acid increased significantly in patients with PBR, which might play an active role in gallstone formation.

PLA2 plays a crucial role in arachidonic acid metabolism and secretion and is upregulated in patients with multiple cholesterol stones [40]. After pancreatin reflux in the biliary tract, the concentration of PLA2 in bile increased. Some studies have shown that arachidonic acid damages the gallbladder mucosa, causing the high secretion of mucin, which plays an important role in stone formation [23, 41]. The present results showed that arachidonic acid increased significantly in the bile of patients with PBR. Therefore, arachidonic acid metabolism may play an active role in gallstone formation in patients with PBR.

Phenylalanine is an essential amino acid that participates in the synthesis of important neurotransmitters and hormones, including tyrosine, and in glucose metabolism and fat metabolism [42]. A study showed that the abnormal proliferation of malignant tumors in the early and middle stages of lung cancer caused normal cell stress reactions, resulting in a significant increase in tyrosine concentration [43]. Other studies have shown that a diet restricted with phenylalanine inhibits the growth and metastasis of several malignancies [44]. We found that phenylalanine and tyrosine in the bile of patients with PBR increased significantly. The GBC incidence in patients with PBR in Japan is 200 times higher than that in patients with non-PBM [45]. We believe that a significant increase in phenylalanine and tyrosine may be related to the mechanism of biliary carcinogenesis in patients with PBR. The present results are consistent with those of a bile metabolomic study on biliary tract cancer [25].

Here, we performed bile metabolomic analysis to compare bile metabolites between patients with PBR and control individuals. To the best of our knowledge, this is the first study on the bile metabolomic analysis of PBR, which is a novelstudy with clinical application value. We found that bile lithogenic and carcinogenic metabolites were significantly different between the PBR and control groups, which would help identify high-risk groups for benign and malignant biliary diseases. However, the present study has some limitations. First, the number of patients included is relatively small. Hence, we are planning to perform a validation study consisting of more bile samples of PBR with gallstones. Second, this is a preliminary study. Nevertheless, the results provide a basis for further studies on the mechanism of gallstone formation in PBR.

## Conclusion

The results indicated that LC–MS-based metabolomics was an effective and promising approach for identifying patients with PBR and bile-specific metabolites, which might help elucidate the PBR mechanism that leads to gallstone formation. Bile metabolites in patients with PBR changed significantly compared with those in the control group. Therefore, patients with PBR can be considered high-risk groups for gallstone formation.

### Electronic supplementary material

Below is the link to the electronic supplementary material.

Supplementary Material 1

## Data Availability

The datasets used and/or analysed during the current study are available from the corresponding author on reasonable request.

## Abbreviations

PBR: Pancreaticobiliary reflux
PBM: Pancreaticobiliary maljunction
PLA2: phospholipase A2
PC: Phosphatidylcholine
lysoPC: Lysophosphatidylcholine
FFAs: Free fatty acids
GBC: Gallbladder carcinoma
PCA: principal component analysis
OPLS-DA: orthogonal projections to latent structure-discriminant analysis
