# Supplementary material for: Identification of changes in bile composition in pancreaticobiliary reflux based on liquid chromatography/mass spectrometry metabolomics
Source: BMC Gastroenterol. 2024 Jan 2;24:5. doi: 10.1186/s12876-023-03097-4 (PMC10759582; doi:10.1186/s12876-023-03097-4)
Supplement: Supplementary file 1 — Supplementary Material 1 [file 12876_2023_3097_MOESM1_ESM.docx]

**Supplementary Data**

**Supplementary table 1.** Significant metabolites in bile samples

| NO. | RT med | MS2 name | mode | M/zmed | VIP | Trend |
| --- | --- | --- | --- | --- | --- | --- |
| 1 | 30.606 | DG(16:1(9Z)/22:5(4Z,7Z,10Z,13Z,16Z)/0:0) | + | 641.516 | 3.204 | ↓ |
| 2 | 30.6688 | DG(20:3(8Z,11Z,14Z)/16:1(9Z)/0:0) | + | 617.514 | 2.859 | ↓ |
| 3 | 40.661 | Palmitic acid | _ | 255.233 | 2.693 | ↑ |
| 4 | 439.545 | N2-Succinoylarginine | + | 275.135 | 2.645 | ↑ |
| 5 | 206.625 | Bovinic acid | _ | 279.233 | 2.488 | ↑ |
| 6 | 206.625 | PC(20:3(8Z,11Z,14Z)/14:0) | + | 279.233 | 2.477 | ↓ |
| 7 | 33.9117 | Physalin L | + | 529.209 | 2.324 | ↑ |
| 8 | 34.481 | Gamma-Linolenic acid | + | 162.055 | 2.295 | ↑ |
| 9 | 366.887 | 3,7-Dimethyluric acid | + | 195.051 | 2.276 | ↑ |
| 10 | 41.889 | Gamma-Linolenic acid | − | 277.217 | 2.261 | ↑ |
| 11 | 39.872 | Docosahexaenoic acid | _ | 327.233 | 2.249 | ↑ |
| 12 | 605.79 | L-Arginine | + | 175.119 | 2.223 | ↑ |
| 13 | 294.163 | L-Leucine | + | 132.102 | 2.186 | ↑ |
| 14 | 338.117 | L-prolyl-L-proline | + | 213.123 | 2.184 | ↑ |
| 15 | 493.961 | L-Histidine | + | 156.077 | 2.161 | ↑ |
| 16 | 331.673 | L-Proline | + | 116.071 | 2.158 | ↑ |
| 17 | 410.19 | L-Glutamic acid | _ | 146.045 | 2.154 | ↑ |
| 18 | 603.563 | L-Lysine | + | 147.113 | 2.128 | ↑ |
| 19 | 294.823 | Piperidine | + | 86.097 | 2.106 | ↑ |
| 20 | 410.183 | Pyrrolidonecarboxylic acid | _ | 128.034 | 2.098 | ↑ |
| 21 | 351.273 | Thiomorpholine 3-carboxylate | + | 148.043 | 2.078 | ↑ |
| 22 | 38.972 | Arachidonic acid | _ | 303.234 | 2.074 | ↑ |
| 23 | 170.129 | PC(20:4(5Z,8Z,11Z,14Z)/14:0) | + | 754.541 | 2.063 | ↓ |
| 24 | 221.128 | LysoPC(16:0) | + | 496.340 | 2.061 | ↑ |
| 25 | 306.114 | L-Methionine | + | 150.059 | 2.06 | ↑ |
| 26 | 51.816 | Pelargonic acid | _ | 157.123 | 2.047 | ↑ |
| 27 | 322.827 | L-Tyrosine | + | 182.081 | 2.026 | ↑ |
| 28 | 373.468 | Oxoglutaric acid | + | 145.013 | 2.025 | ↑ |
| 29 | 330.05 | L-Proline | _ | 114.055 | 2.021 | ↑ |
| 30 | 291.176 | L-Norleucine | _ | 130.086 | 2.02 | ↑ |
| 31 | 188.862 | Sulfolithocholylglycine | + | 514.283 | 2.014 | ↑ |
| 32 | 382.216 | Sarcosine | + | 90.0554 | 2.001 | ↑ |
| 33 | 309.039 | N-Acetylserine | _ | 146.045 | 2.001 | ↑ |
| 34 | 35.286 | Physalin D | + | 545.204 | 1.999 | ↑ |
| 35 | 222.45 | LysoPC(16:1(9Z)/0:0) | + | 494.325 | 1.997 | ↑ |
| 36 | 374.847 | L-Threonine | _ | 118.05 | 1.991 | ↑ |
| 37 | 223.306 | LysoPC(15:0) | + | 482.324 | 1.983 | ↑ |
| 38 | 279.965 | L-Phenylalanine | _ | 166.086 | 1.983 | ↑ |
| 39 | 395.502 | Beta-Guanidinopropionic acid | + | 130.061 | 1.980 | ↑ |
| 40 | 304.071 | Racemethionine | + | 148.043 | 1.972 | ↑ |
